# Impact of delirium in critically ill patients on long-term health-related quality of life and cognitive functioning

**DOI:** 10.1186/cc9758

**Published:** 2011-03-11

**Authors:** M Van den Boogaard, L Schoonhoven, A Evers, J Van der Hoeven, TH Van Achterberg, P Pickkers

**Affiliations:** 1 Radboud University Nijmegen Medical Centre, Nijmegen, the Netherlands; 2 Scientific Institute for Quality of Healthcare, Radboud University Nijmegen Medical Centre, Nijmegen, the Netherlands; 3 Department of Medical Psychology, Radboud University Nijmegen Medical Centre, Nijmegen, the Netherlands

## Introduction

Delirium is associated with long-term cognitive decline and poor health-related quality of life (HRQOL). Little is known about long-term differences on these aspects between critically ill patients with and without delirium during their ICU stay, differences between delirium subtypes on HRQoL and the effect of delirium duration on HRQoL.

## Methods

At 18 months after ICU discharge an HRQoL survey was sent to 1,292 ICU survivors with (*n *= 272) and without (*n *= 1,020) delirium during their ICU stay. The survey consisted of the Short Form (SF)-36, the Checklist Individual Strength (CIS)-fatigue and the Cognitive Failure Questionnaire (CFQ). Covariance analysis was performed to adjust for gender, sepsis, APACHE II score and length of stay.

## Results

A total of 915 (71%) patients responded, of which 171 patients were delirious during their ICU stay (median age 65 (IQR 58 to 85), APACHE II score 17 (IQR 14 to 20)) and 745 patients were not (median age 65 (IQR 57 to 72), APACHE II score 13 (IQR 10 to 16)). After adjusting for covariates, no differences were found between delirious and nondelirious ICU survivors on the SF-36 and CIS-fatigue. However, delirious ICU survivors were significantly more absent-minded (*P *= 0.02), suffered a more pronounced change in cognitive function compared with prior to their ICU stay (*P *< 0.01), and their total CFQ score was significantly (*P *= 0.03) lower compared with ICU survivors that had not been delirious. Hypoactive delirious survivors performed significantly better on several domains of the SF-36 than mixed and hyperactive delirious patients. Duration of delirium tended to correlate with changed health condition after ICU stay (*r *= -0.15; *P *= 0.06).

## Conclusions

ICU survivors that were delirious during their ICU stay experience significantly more cognitive failure than those who were not, even after adjusting for relevant covariates. Hypoactive delirious patients are less affected compared with other subtypes of delirium. Duration of delirium appears to relate to HRQoL.

